# Evidence for nephrolithiasis preceding sarcoidosis diagnosis: findings from a Swedish case-control study

**DOI:** 10.1186/s12931-026-03877-y

**Published:** 2026-08-28

**Authors:** Joanna Werner, Elizabeth V. Arkema, Tomas Thiel, Nurgul Batyrbekova, Susanna Kullberg

**Affiliations:** 1 Division of Immunology and Respiratory Medicine, Department of Medicine Solna, Karolinska Institutet, Gävlegatan 55, Stockholm, NB3:03, SE-171 76 Sweden; 2 Department of Respiratory Medicine, Theme Inflammation and Ageing, Karolinska University Hospital, Stockholm, Sweden; 3 Division of Clinical Epidemiology, Department of Medicine Solna, Karolinska Institutet, Stockholm, Sweden; 4 Department of Clinical Science, Intervention, and Technology (CLINTEC), Karolinska Institutet, Stockholm, Sweden; 5 Department of Urology, Karolinska University Hospital, Stockholm, Sweden

**Keywords:** Sarcoidosis, Nephrolithiasis, Calcium metabolism

## Abstract

**Background:**

Patients with sarcoidosis have an increased risk for calcium metabolism disturbances, such as hypercalcemia and hypercalciuria. Previous studies suggest that nephrolithiasis (stone formation in the renal and ureter, often caused by dysregulated calcium balance) may occur long before sarcoidosis diagnosis and may be an early sign of not-yet-diagnosed sarcoidosis. We hypothesized that a history of nephrolithiasis is associated with future risk of sarcoidosis.

**Methods:**

In this population-based matched case-control study, we included individuals with sarcoidosis and matched general population controls and identified those with a history of nephrolithiasis in Swedish nationwide registers. Patients with sarcoidosis who received treatment within three months of their sarcoidosis diagnosis were classified as severe. Nephrolithiasis severity was defined based on the need for intervention, multiple stones and early onset. Conditional logistic regression models estimated adjusted odds ratios with 95% confidence intervals (OR; 95%CI).

**Results:**

A total of 10,904 sarcoidosis patients and 99,211 general population controls were included. At sarcoidosis diagnosis, 4.5% of patients had a history of nephrolithiasis compared to 2.6% of controls. Nephrolithiasis was associated with a 61% increased odds of sarcoidosis (adjusted OR = 1.61; 95%CI 1.46–1.78). The OR of severe sarcoidosis associated with a history of severe nephrolithiasis was 1.35 (95%CI 0.58–3.16).

**Conclusions:**

These findings indicate that nephrolithiasis might be an early sign of sarcoidosis in a subset of patients.

**Supplementary Information:**

The online version contains supplementary material available at https://doi.org/10.1186/s12931-026-03877-y.

## Background

Sarcoidosis is an inflammatory disease of unknown etiology, leading to formation of non-necrotizing granulomas, which can affect many different organs in the body. Around 90% of patients show signs of lung parenchymal and/or intrathoracic lymph node involvement [1, 2]. Time from first symptoms to sarcoidosis diagnosis is often delayed and a subclinical phase may be evident up to ten years before diagnosis [3].

It has been known since the 1930s that calcium metabolism disturbances, e.g. hypercalcemia and hypercalciuria, are associated with sarcoidosis [4, 5]. A well-known complication of a dysregulated calcium balance is stone formation in the urinary system (renal and ureter, i.e. nephrolithiasis), where the majority of stones are formed by calcium oxalate [6]. A few smaller studies and case reports have described an association between nephrolithiasis and sarcoidosis, showing that nephrolithiasis can be the presenting symptom of sarcoidosis [7–11].

In this study, we set out to investigate this in a larger population by performing a matched case-control study using Swedish nation-wide registers, which are further described in the Methods section. Our aim was to investigate whether nephrolithiasis is associated with risk of future sarcoidosis and thus might be a sign for not yet diagnosed disease. Furthermore, we sought to explore whether the association differs by severity of sarcoidosis and nephrolithiasis.

## Methods

### Study population

We conducted a matched case-control study using Swedish nationwide registers. Records across registers were linked using each person’s unique identification number, issued to all residents in Sweden. Information on nephrolithiasis, sarcoidosis diagnoses and comorbidities was obtained using International Classification of Diseases (ICD) codes, 10th revision, and Classification of Health Care Measures (KVÅ) codes, for diagnoses and interventional procedures (see supplemental table S1 for codes) from the National Patient Register (NPR). The NPR captures information on hospitalizations since 1967 (full coverage from 1987), and from outpatient (non-primary care) visits from 2001. Sarcoidosis cases were identified from the NPR and required to have at least two inpatient or outpatient visits listing an ICD-10 code for sarcoidosis (D86) between 2008 and 2023, while individuals with any sarcoidosis visit before 2008 were excluded.

Up to 10 general population controls were randomly sampled from the Total Population Register (TPR) and matched to each case on age, sex and residential location. They were required to be living in Sweden, with no history of sarcoidosis, at the time of the matched case’s first sarcoidosis diagnosis code (index date).

The study population was restricted to individuals aged 18–85 years at index date. We excluded individuals who emigrated or were deceased prior to index date. Furthermore, we also excluded individuals registered in the Swedish Cancer Register with a former or present malignancy (see supplemental table S1) due to risk of sarcoidosis misclassification and since malignancies are associated with a risk of hypercalcemia, which can cause nephrolithiasis.

### Nephrolithiasis

Nephrolithiasis was defined as any inpatient or outpatient visit in the NPR with an ICD-10 code for nephrolithiasis (N20) listed as a primary or secondary diagnosis. Individuals were considered to have a history of nephrolithiasis if they had any visit in the NPR listing nephrolithiasis before the index date.

There is no established definition of severe nephrolithiasis. Treatment options depend on stone size. Most renal stones pass spontaneously or with the help of pain killers and spasmolytic drugs [12, 13]. Larger stones, over 2 cm, or failure of other interventions may require percutaneous nephrolithotomy (PNCL) [14]. A recurrent renal stone within five years [15–17] and young age [16] are risk factors for recurrent renal stones. To further reduce recurrence risk, potassium citrate can be prescribed [14]. Therefore, severe nephrolithiasis was defined as meeting at least one of the following criteria: (1) intervention with percutaneous nephrolithotomy (PNCL); (2) multiple stones, defined as > 1 stone during a 5-year period, with every stone being > 1 year apart; (3) stone formation before age 25; (4) prescription of potassium citrate. Information on prescribed medications were extracted from the Prescription Drug Register (PDR) using Anatomical Therapeutic Chemical (ATC) codes (see supplemental table S1 for codes). The PDR started in 2005, which allows for information on prescribed medications to be collected at least three years prior to inclusion.

### Other variables

Demographic variables were evaluated at the index date using data obtained from the TPR. These included age, sex, region of residence (grouped into six healthcare regions), country of birth (Nordic, non-Nordic or missing), and attained education (< 10 years, 10–12 years, > 12 years or missing).

Severe sarcoidosis was defined as at least one prescription of systemic corticosteroids, methotrexate or azathioprine in the PDR within ± 3 months from sarcoidosis diagnosis (see codes in supplemental table S1). This definition of severe sarcoidosis has been shown associated with increased mortality in sarcoidosis [18].

Diagnoses associated with both sarcoidosis and nephrolithiasis were considered as potential confounders and identified using data on healthcare visits in the NPR and/or medication dispensations in the PDR (see supplemental table S1 for codes). We chose to only include diagnoses where there is sufficient evidence for their association with both sarcoidosis and nephrolithiasis. The following diagnosis groups were defined: “autoimmune diseases” consisting of inflammatory bowel disease [19, 20] and ankylosing spondylitis [21, 22], “renal insufficiency or failure” consisting of renal insufficiency and renal failure [23, 24], “metabolic syndrome related diseases” consisting of bariatric surgery [25, 26], type 2 diabetes, diabetes associated with pregnancy and dispensed prescription medication for type 2 diabetes [27, 28], and “osteoporosis” consisting of osteoporosis diagnosis [29, 30]. Bariatric surgery was chosen as a proxy for severe obesity [31], as data on body mass index were not available; however, bariatric surgery has also itself been shown to increase the risk for nephrolithiasis [25].

### Statistical analysis

All analyses were stratified by sex, age and region of residence to assess whether these variables acted as effect modifiers. Using descriptive statistics, we compared data within different stratifications. Categorical variables were presented as counts and proportions, and continuous variables as means with standard deviations (SD).

Conditional logistic regression was used to account for the matched case-control design and estimate odds ratios (ORs) with 95% confidence intervals (CIs) for the association between nephrolithiasis and sarcoidosis. Separate models were fitted for any nephrolithiasis and severe nephrolithiasis. Each model was adjusted for the following diagnosis groups: autoimmune comorbidities, renal insufficiency or failure, metabolic syndrome related diseases, and osteoporosis.

Because renal insufficiency/failure, metabolic syndrome related diseases and osteoporosis could be consequences of undiagnosed sarcoidosis rather than risk factors for nephrolithiasis, we conducted a sensitivity analysis which adjusted for these conditions only if they occurred at least 5 years before the index date. Diagnoses recorded more than 5 years before the index date, but after nephrolithiasis, were not considered as confounders in these models. No time restriction was applied to inflammatory bowel disease and ankylosing spondylitis, as autoimmune conditions typically have a long preclinical phase.

We used statistical software R version 4.3.2 (R Foundation for Statistical Computing, Vienna, Austria) for data management and analyses.

Ethical approval was obtained from the Regional Ethical Review Board in Stockholm (DNR: 2014/230-31, 2021-01856).

## Results

**Fig. 1:**
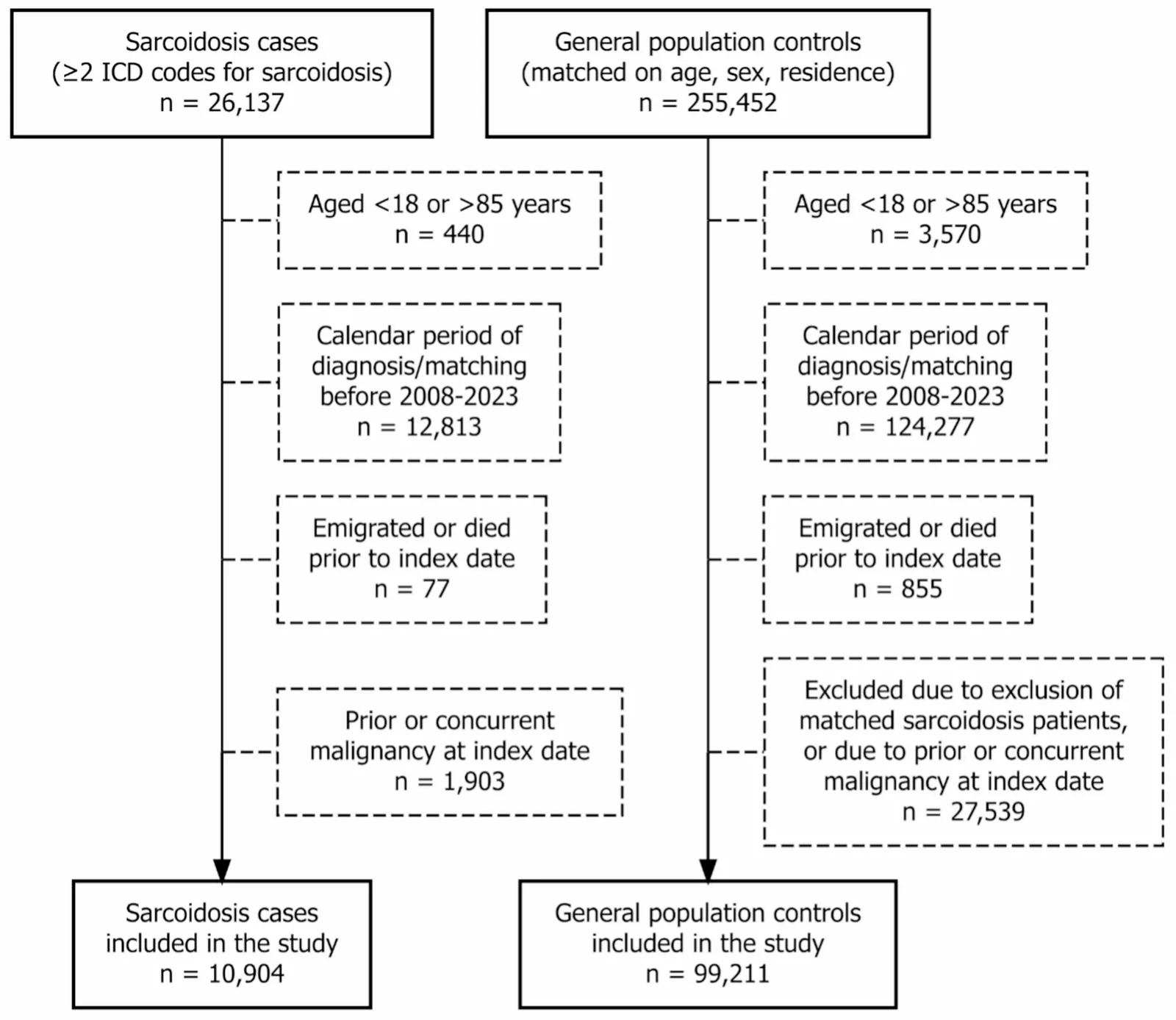
Selection of sarcoidosis patients and general population controls. Sarcoidosis cases were diagnosed between 2008 and 2023, and general population controls matched on age, sex, and county of residence

This study included 10,904 sarcoidosis patients and 99,211 matched general population controls (Fig. 1). The median age at index date was approximately 50 years. The majority were male (60.0% of sarcoidosis patients, 61.6% of controls) and born in a Nordic country (87.3% of sarcoidosis patients, 82.9% of controls). A lower proportion of sarcoidosis patients had > 12 years of education compared to controls (32.0% vs. 36.3%). Among sarcoidosis patients, 43.1% received treatment around the time of diagnosis. Detailed characteristics are shown in Table 1.

**Table 1:** Clinical characteristics of sarcoidosis patients and controls

	All sarcoidosis (*N* = 10904)	Controls (*N* = 99211)	Severe Sarcoidosis^†^ (*N* = 4697)	Controls (*N* = 42896)	Non-severe sarcoidosis (*N* = 6207)	Controls (*N* = 56315)
Age at the index date						
Median (Q1, Q3)	50.6 (40.1, 61.4)	49.4 (39.2, 59.7)	50.4 (39.3, 61.2)	49.0 (38.6, 59.6)	50.7 (40.6, 61.4)	49.6 (39.7, 59.7)
Mean (SD)	51.0 (14.2)	49.9 (13.7)	50.7 (14.5)	49.6 (14.1)	51.2 (13.9)	50.1 (13.5)
Sex						
Male	6545 (60.0%)	61,082 (61.6%)	2870 (61.1%)	26,820 (62.5%)	3675 (59.2%)	34,262 (60.8%)
Female	4359 (40.0%)	38,129 (38.4%)	1827 (38.9%)	16,076 (37.5%)	2532 (40.8%)	22,053 (39.2%)
Nephrolithiasis						
Yes	496 (4.5%)	2591 (2.6%)	215 (4.6%)	1096 (2.6%)	281 (4.5%)	1495 (2.7%)
No	10,408 (95.5%)	96,620 (97.4%)	4482 (95.4%)	41,800 (97.4%)	5926 (95.5%)	54,820 (97.3%)
Country of birth						
Nordic*	9514 (87.3%)	82,215 (82.9%)	4149 (88.3%)	35,758 (83.4%)	5365 (86.4%)	46,457 (82.5%)
Non-Nordic	1389 (12.7%)	16,989 (17.1%)	548 (11.7%)	7136 (16.6%)	841 (13.5%)	9853 (17.5%)
Missing	1 (0.0%)	7 (0.0%)	0 (0.0%)	2 (0.0%)	1 (0.0%)	5 (0.0%)
Region						
Stockholm	2292 (21.0%)	20,971 (21.1%)	742 (15.8%)	6819 (15.9%)	1550 (25.0%)	14,152 (25.1%)
Uppsala-Örebro	2325 (21.3%)	21,380 (21.6%)	1096 (23.3%)	10,086 (23.5%)	1229 (19.8%)	11,294 (20.1%)
West	1895 (17.4%)	17,166 (17.3%)	849 (18.1%)	7719 (18.0%)	1046 (16.9%)	9447 (16.8%)
South	1943 (17.8%)	17,514 (17.7%)	914 (19.5%)	8298 (19.3%)	1029 (16.6%)	9216 (16.4%)
Southeast	1279 (11.7%)	11,580 (11.7%)	621 (13.2%)	5636 (13.1%)	658 (10.6%)	5944 (10.6%)
North	1170 (10.7%)	10,600 (10.7%)	475 (10.1%)	4338 (10.1%)	695 (11.2%)	6262 (11.1%)
Education						
< 10 yrs	1858 (17.0%)	16,026 (16.2%)	841 (17.9%)	7141 (16.6%)	1017 (16.4%)	8885 (15.8%)
10–12 yrs	5437 (49.9%)	45,568 (45.9%)	2401 (51.1%)	20,057 (46.8%)	3036 (48.9%)	25,511 (45.3%)
> 12 yrs	3490 (32.0%)	36,033 (36.3%)	1414 (30.1%)	15,042 (35.1%)	2076 (33.4%)	20,991 (37.3%)
Missing	119 (1.1%)	1584 (1.6%)	41 (0.9%)	656 (1.5%)	78 (1.3%)	928 (1.6%)

Characteristics of sarcoidosis patients diagnosed between 2008 and 2023 and general population controls matched on age, sex, and county of residence. Sarcoidosis patients were stratified on severity^†^

*Nordic countries include Sweden, Denmark, Norway, Finland and Iceland

^†^Sarcoidosis was defined as severe if the patient received immunosuppressive treatment within ± 3 months of diagnosis

### Association between sarcoidosis and nephrolithiasis

History of nephrolithiasis was more frequent among sarcoidosis patients compared with general population controls (4.5% vs. 2.6%, Table 1) and was associated with increased odds of sarcoidosis (adjusted OR (aOR) = 1.61; 95% CI 1.46–1.78, Table 2). The aOR for severe sarcoidosis (aOR = 1.62; 95% CI 1.38, 1.90, Table 2) was similar to the aOR for non-severe sarcoidosis (aOR = 1.62; 95% CI 1.42, 1.85, Table 2). The median time between nephrolithiasis and sarcoidosis diagnosis was approximately 5 years in both severe and non-severe sarcoidosis patients (Supplementary table S2). History of severe nephrolithiasis showed a positive but imprecise association with severe sarcoidosis (unadjusted OR = 1.35; 95% CI 0.58–3.16; Table 2). Due to low counts, adjusted estimates for severe nephrolithiasis could not be calculated. The aORs for severe nephrolithiasis and overall sarcoidosis (aOR = 1.15; 95% CI 0.59, 2.27), as well as between severe nephrolithiasis and non-severe sarcoidosis (aOR = 1.00; 95% CI 0.38, 2.63), did not show any associations (Table 2).

**Table 2:** Association between history of nephrolithiasis and risk of sarcoidosis

	Sarcoidosis vs. Controls				Severe sarcoidosis^†^ vs. Controls				Non-severe sarcoidosis vs. Controls			
	Cases *N*	Controls *N*	OR (95% CI)	Adjusted OR* (95% CI)	Cases *N*	Controls *N*	OR (95% CI)	Adjusted OR* (95% CI)	Cases *N*	Controls *N*	OR (95% CI)	Adjusted OR* (95% CI)
Nephrolithiasis												
No	10,408	96,620	1 (Ref)	1 (Ref)	4482	41,800	1 (Ref)	1 (Ref)	5926	54,820	1 (Ref)	1 (Ref)
Yes	496	2591	1.75 (1.59, 1.94)	1.61 (1.46, 1.78)	215	1096	1.82 (1.56, 2.11)	1.62 (1.38, 1.90)	281	1495	1.71 (1.50, 1.95)	1.62 (1.42, 1.85)
Severe Nephrolithiasis**												
No	419	2188	1 (Ref)	1 (Ref)	180	936	1 (Ref)	insufficient	239	1252	1 (Ref)	1 (Ref)
Yes	77	403	1.13 (0.61, 2.11)	1.15 (0.59, 2.27)	35	160	1.35 (0.58, 3.16)	counts	42	243	0.93 (0.38, 2.31)	1.00 (0.38, 2.63)

Adjusted odds ratios and 95% confidence intervals (OR 95% CI) for the association between history of nephrolithiasis and risk of sarcoidosis. General population controls were matched to sarcoidosis patients on age, sex, and county of residence

*Odds ratio adjusted for history of autoimmune diseases, renal insufficiency or failure, metabolic diseases, and osteoporosis

^†^Sarcoidosis was defined as severe if the patient received immunosuppressive treatment within ± 3 months of diagnosis

**Severe nephrolithiasis was defined as meeting at least one of the following criteria: (1) intervention with percutaneous nephrolithotomy (PNCL); (2) multiple stones, defined as > 1 stone during a 5-year period, with every stone being > 1 year apart; (3) stone formation before age 25; (4) prescription of potassium citrate

### Stratified analyses by age, sex, and region

**Fig. 2:**
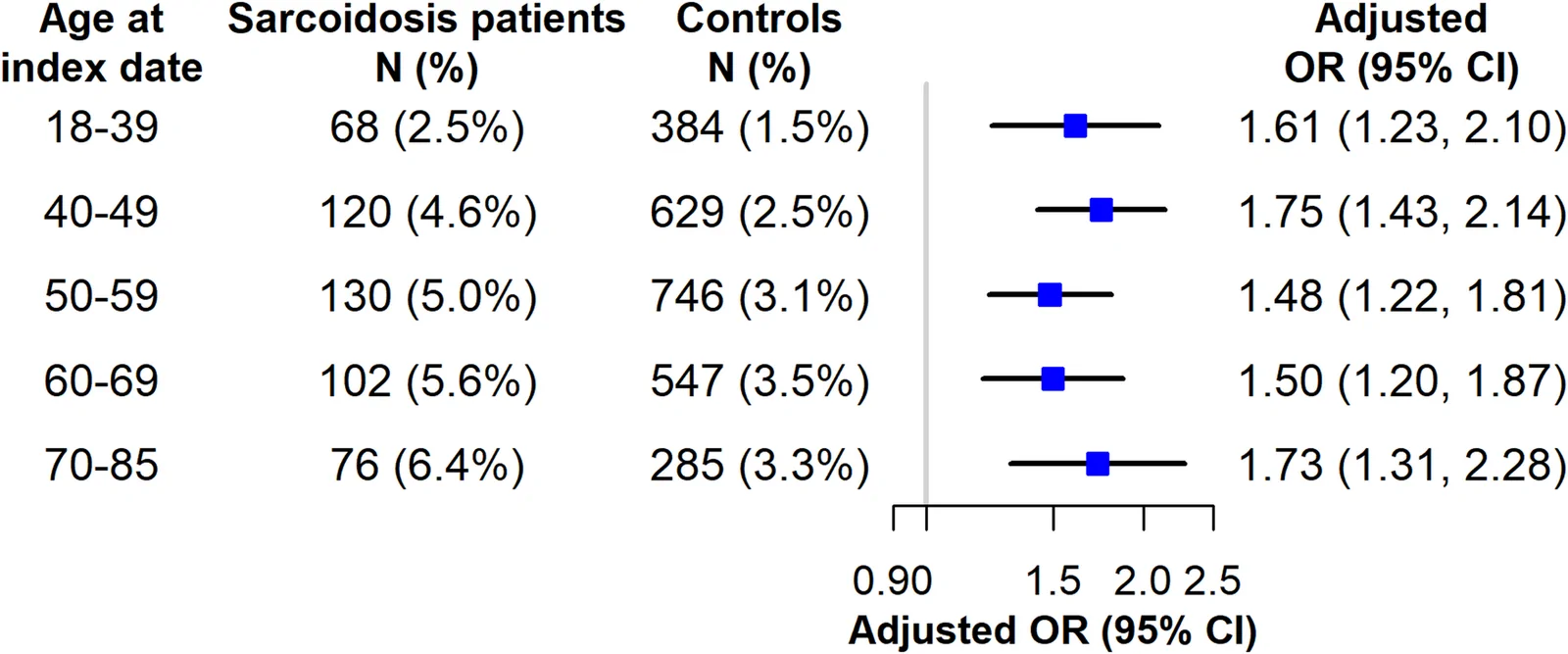
Association between history of nephrolithiasis and risk of sarcoidosis, stratified by age. Numbers and percentages of sarcoidosis cases and general population controls with nephrolithiasis before diagnosis or matching date. Adjusted odds ratios* and confidence intervals (aOR CI) for the association between a history of nephrolithiasis and incident sarcoidosis. *Odds ratios adjusted for history of autoimmune diseases, renal insufficiency or failure, metabolic diseases, and osteoporosis

**Fig. 3:**
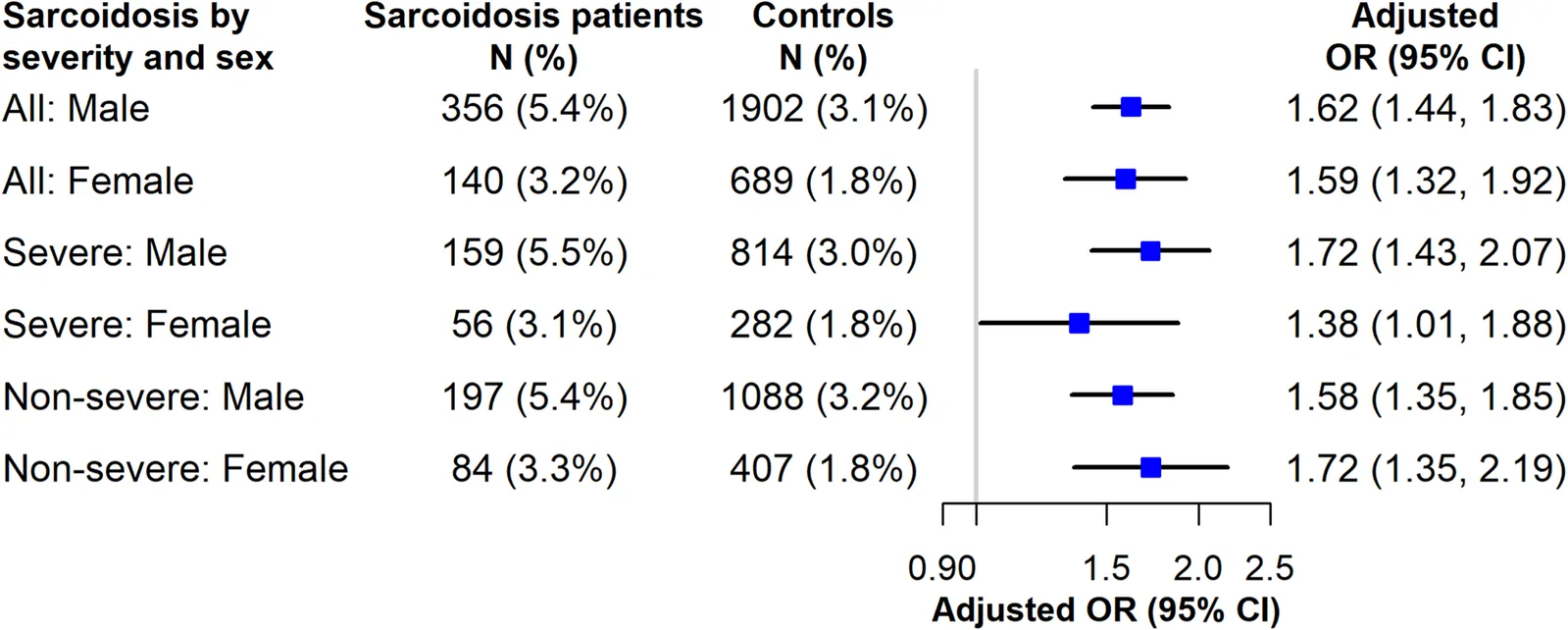
Association between history of nephrolithiasis and risk of sarcoidosis, stratified by sarcoidosis severity† and sex. Numbers and percentages of sarcoidosis cases and general population controls with nephrolithiasis before diagnosis or matching date. Adjusted odds ratios* and confidence intervals (aOR CI) for the association between a history of nephrolithiasis before sarcoidosis diagnosis and incident sarcoidosis. *Odds ratios adjusted for history of autoimmune diseases, renal insufficiency or failure, metabolic diseases, and osteoporosis. ^†^Sarcoidosis was defined as severe if the patient received immunosuppressive treatment within ± 3 months of diagnosis

When stratifying by age at index date, the association between a history of nephrolithiasis and sarcoidosis was strongest in groups aged 40–49 and 70–85 years (Fig. 2, Supplemental S3). ORs differed by sex and sarcoidosis severity. However, the differences were imprecise with partly overlapping CIs. The most pronounced difference was the association with nephrolithiasis and severe sarcoidosis with higher aOR in males (aOR = 1.72; 95% CI 1.43–2.07) than in females (aOR = 1.38; 95% CI 1.01–1.88, Fig. 3, Supplemental S3). For non-severe sarcoidosis, the association was slightly higher in females (aOR = 1.72; 95% CI 1.35–2.19) compared with males (aOR = 1.58; 95% CI 1.35–1.85, Fig. 3, Supplemental S3). Residential location appeared to influence the association, with higher ORs for sarcoidosis associated with nephrolithiasis among individuals residing in the Uppsala–Örebro, West, and Southeast regions compared with other regions (Supplemental S3).

Formal interaction analyses were not performed due to low counts in these subgroups, and results are to be interpreted as exploratory.

Results of sensitivity analyses which only considered comorbidities diagnosed more than 5 years before the index date (except for autoimmune disorders) were similar to the main results (Supplementary table S4). Results were also similar when stratifying by age, sex and region of residence (Supplementary table S5).

## Discussion

In this population-based nationwide study, a history of nephrolithiasis was associated with 61% higher odds of sarcoidosis. Thus, nephrolithiasis in some cases might be caused by underlying sarcoidosis. This is, to our knowledge, the largest investigation on nephrolithiasis and sarcoidosis, extending previous evidence from smaller clinical investigations [8, 9, 11] and case reports [7, 10]. Two recent studies reported similar results, one study from Brazil consisting of mainly female sarcoidosis patients [32] and an epidemiological study on black women in the United States [33], supporting the robustness of our findings and their potential generalizability to other populations.

Our finding that nephrolithiasis occurs more frequently than expected several years before sarcoidosis diagnosis is in line with previous studies showing that symptoms of sarcoidosis including calcium metabolism disturbances can appear years before diagnosis [5, 34], and that there is an increase in inflammatory plasma protein levels more than ten years preceding sarcoidosis diagnosis [3]. Among these increased proteins are chemokine ligand 3 (CCL-3) and tumor necrosis factor-alpha (TNF$\:\alpha\:)$, both involved in calcium metabolism by promoting bone resorption through the activation of osteoclasts [35, 36].

Interestingly, sarcoidosis-associated calcium metabolism disturbances, i.e. hypercalcemia, is more frequent in patients with multiorgan involvement and development of chronic disease [37], and an indication to initiate treatment with corticosteroids [38]. We did not see any differences in the ORs between all nephrolithiasis and sarcoidosis severity. Although weak with an unadjusted OR of 1.35 and a broad CI, we believe that the association between severe nephrolithiasis and subsequent development of severe sarcoidosis is worth noting and might lend further support to the association between calcium metabolism disturbances including severe nephrolithiasis and potentially a more severe disease course.

Apart from being associated with a more severe disease course, hypercalcemia and hypercalciuria, which most likely predisposes nephrolithiasis, have both been associated with male sex [1, 39, 40]. One study showed that males were younger at sarcoidosis diagnosis, more often had a more severe radiographic stage and presented with hypercalcemia almost three times as often as females [41]. Therefore, our observation of a higher OR for nephrolithiasis and severe sarcoidosis in males compared to females is interesting, although the difference was small with partly overlapping confidence intervals. Differences in age is another factor to consider. In our study, the most profound differences between sarcoidosis patients and controls were in age groups 40–49 years old and 70–85 years old, although this should be interpreted with caution due to low numbers. Hormonal factors seem to influence the risk for sarcoidosis [42] as well as for nephrolithiasis [43] and sarcoidosis symptoms differ between sexes [44] and age [41]. There may be different environmental exposures and dietary habits between men and women as well as between different ages, influencing sarcoidosis severity. Recently, the contribution of vitamins, including vitamin K, to nephrolithiasis has received increasing attention. Polymorphism of the vitamin K epoxide reductase complex subunit 1, which plays a crucial role in vitamin K metabolism and implicated in calcification processes, was associated with nephrolithiasis in sarcoidosis [45].

Water hardness, a measure of the concentration of dissolved minerals, primarily calcium [46] varies around Sweden [47] and we observed variation in prevalence of nephrolithiasis among regions. Previous studies on water hardness and nephrolithiasis have yielded conflicting results, partly due to the complexity of mineral interactions [48]. Individuals with a dysregulated calcium metabolism, as in sarcoidosis, may be more sensitive to external factors, such as minerals in tap water, though this warrants further investigation.

This study has some limitations. Due to the structure of the available data, a case-control design nested within Swedish registers was used. This design yields effect estimates equivalent to those from a cohort study under these conditions. Sarcoidosis cases were identified using ICD-10 codes, and some misclassification is possible. Nevertheless, this misclassification is likely minimal, as a previous validation study showed that ICD-10 codes have a high positive predictive value for sarcoidosis [49]. Nephrolithiasis cases were also identified through ICD codes and to our knowledge, the ICD-10 codes have not been validated in Swedish registers. However, a study from the U.S. showed ICD-9 codes to accurately capture nephrolithiasis [50]. Furthermore, we are missing minor renal stones if patients did not seek healthcare or if they were diagnosed in primary care, however, we believe that this misclassification affects sarcoidosis cases and controls equally.

Another limitation is the use of proxies for severe sarcoidosis and severe nephrolithiasis. There is no defined classification system describing severity for any of these conditions [51, 52]. Previous research has shown that sarcoidosis patients with an indication for systemic treatment around diagnosis have a two-fold increased risk of death [18]. However, we cannot rule out the possibility that initiating treatment might have been influenced by local prescribing patterns and not only by disease severity or symptoms.

We were unable to evaluate the composition of the renal stones. Although more than 80% of all renal stones are made up of calcium [53] and the main risk factor for renal stones is hypercalciuria [54], we could not determine whether the higher proportion of renal stones observed in sarcoidosis patients was caused by calcium dysregulation or by other factors. We lacked information on risk factors for nephrolithiasis such as dietary and lifestyle factors and we cannot rule out the possibility of residual confounding from unmeasured factors. We could not fully adjust for obesity which is a risk factor for nephrolithiasis [55, 56] and sarcoidosis [26, 57]. However, we adjusted for bariatric surgery and type 2 diabetes, which should have captured at least a proportion of individuals with obesity and the metabolic syndrome, both well-known risk factors for nephrolithiasis [55]. Nevertheless, the risk of confounding from metabolic and lifestyle factors must be considered, as well as unmeasured confounders.

A major strength of our study is the excellent coverage and validity of Sweden’s population-based registers leading to little or no loss to follow-up [58]. The prospective nature of these registers enabled us to identify nephrolithiasis diagnoses before sarcoidosis. The overall study was adequately powered; however, subgroup analyses were likely underpowered. In addition, we were able to adjust for many known confounders for renal stones, thus increasing the reliability of our results. We believe that our results can be generalized to Nordic populations. Besides studying nephrolithiasis in relation to sarcoidosis, this study also provides valuable information on the prevalence of nephrolithiasis in Sweden in the general population which has not been studied before.

In conclusion, the results from this study show that nephrolithiasis might represent an early manifestation of sarcoidosis. Individuals with a history of nephrolithiasis had 61% higher odds of sarcoidosis than those without. In some patients with nephrolithiasis, underlying sarcoidosis might be considered as a potential cause.

## Supplementary Information

Supplementary Material 1.

## Data Availability

The datasets used to perform this study are covered by ethics and secrecy agreements and are not publicly available.

## Abbreviations

ICD: International Classification of Diseases
KVÅ: Classification of Health Care Measures
NPR: National Patient Register
PDR: Prescription Drug Register
ATC: Anatomical Therapeutic Chemical
TPR: Total Population Register
PNCL: Percutaneous nephrolithotomy
SD: Standard Deviation
OR: Odds Ratio
CI: Confidence Interval
CCL-3: Chemokine ligand 3
TNF$\:\alpha\:$: Tumor necrosis factor-alpha
