# Supplementary material for: Evidence for nephrolithiasis preceding sarcoidosis diagnosis: findings from a Swedish case-control study
Source: Respir Res. 2026 Aug 28;27:345. doi: 10.1186/s12931-026-03877-y (PMC13548631; doi:10.1186/s12931-026-03877-y)
Supplement: Supplementary file 1 [file 12931_2026_3877_MOESM1_ESM.docx]

**Supplement**

**Evidence for nephrolithiasis preceding sarcoidosis diagnosis: Findings from a Swedish case-control study**

Joanna Werner, Elizabeth V. Arkema, Tomas Thiel, Nurgul Batyrbekova, Susanna Kullberg

| **Table S1**: ICD-7, ICD-10, ATC and KVÅ codes* used to define study population | |
| --- | --- |
| **Diagnoses** | **ICD-10 codes** |
| Sarcoidosis | D86 |
| Nephrolithiasis | N20 |
| **Diagnoses excluded** | **ICD-7 and ICD-10 codes** |
| Malignant tumors | ICD-7 140-209, ICD-10 C00-C97, D45, D471, D473, D474 |
| **Diagnoses adjusted for** | **ICD-10 codes** |
| Autoimmune diseases | M45, K50-51 |
| Renal insufficiency and renal failure | [E10.2C, E11.2C, I12.0, I12.9, I13.1, I13.2, N17, N18, N19.9, N28.0, N99.0, O90.4](https://verktyg.internetmedicin.se/koder/diagnos/e00_e90/e10_e14/e10/e10__2c-diabetes-mellitus-typ-1-med-grav-njurinsufficiens) |
| Metabolic syndrome related diseases | JDF, E11-14, O24.1, O24.2, O24.4, O24.9 |
| Osteoporosis | M80-M82 |
| **Medication used in definition of severe sarcoidosis** | **ATC codes** |
| Corticosteroids | H02AB |
| Methotrexate | L01BA01, L04AX03 |
| Azathioprine | L04AX01 |
| **Procedure used in definition of severe nephrolithiasis** | **KVÅ code** |
| Percutaneous Nephrolithotomy | KAE11 |
| **Medication adjusted for (in group "Metabolic syndrome related diseases")** | **ATC code** |
| Blood glucose lowering drug excluding insulin | A10B |
| *ICD-10 = International Classification of Diseases, 10th revision, codes used in Swedish Cancer Register since 1997, ICD-7 = International Classification of Diseases, 7th revision, codes used in Swedish Cancer Register since 1958, ATC = Anatomical Therapeutic Chemical Classification,  KVÅ = Klassifikation av vårdåtgärder, English: Classification of Health Care Measures | |

**Table S2**. Time in years from nephrolithiasis to sarcoidosis diagnosis. Sarcoidosis patients where stratified on severity^†^.

|  | **All sarcoidosis** | **Severe sarcoidosis**^†^ | **Non-severe sarcoidosis** |
| --- | --- | --- | --- |
| Time from nephrolithiasis* | 5.02 (1.66 - 9.67) | 4.92 (1.33 - 9.23) | 5.03 (1.79 - 9.82) |

*Time in years, median (Q1-Q3).

^†^Sarcoidosis was defined as severe if the patient received immunosuppressive treatment within ± 3 months of diagnosis.

**Table S3:** Odds ratios and 95% confidence intervals (OR 95% CI) for the association between history of nephrolithiasis and sarcoidosis, stratified by age at index date, sex and residential location. General population controls were matched to sarcoidosis patients on age, sex, calendar year and county of residence.

|  |  | **All sarcoidosis vs Controls** | | | | **Severe sarcoidosis**^†^ **vs Controls** | | | | **Non-severe sarcoidosis vs Controls** | | | |
| --- | --- | --- | --- | --- | --- | --- | --- | --- | --- | --- | --- | --- | --- |
|  |  | **Cases N** | **Controls N** | **OR (95% CI)** | **Adjusted OR* (95% CI)** | **Cases N** | **Controls N** | **OR (95% CI)** | **Adjusted OR* (95% CI)** | **Cases N** | **Controls N** | **OR (95% CI)** | **Adjusted OR* (95% CI)** |
| **Age at the index date** |  |  |  |  |  |  |  |  |  |  |  |  |  |
| 18-39 | Nephrolithiasis |  |  |  |  |  |  |  |  |  |  |  |  |
|  | No | 2646 | 26020 | 1 (Ref) | 1 (Ref) | 1203 | 11889 | 1 (Ref) | 1 (Ref) | 1443 | 14131 | 1 (Ref) | 1 (Ref) |
|  | Yes | 68 | 384 | 1.73 (1.33, 2.25) | 1.61 (1.23, 2.10) | 29 | 159 | 1.79 (1.20, 2.69) | 1.64 (1.08, 2.48) | 39 | 225 | 1.69 (1.19, 2.39) | 1.60 (1.13, 2.27) |
| 40-49 | Nephrolithiasis |  |  |  |  |  |  |  |  |  |  |  |  |
|  | No | 2463 | 24068 | 1 (Ref) | 1 (Ref) | 1025 | 9999 | 1 (Ref) | 1 (Ref) | 1438 | 14069 | 1 (Ref) | 1 (Ref) |
|  | Yes | 120 | 629 | 1.84 (1.50, 2.25) | 1.75 (1.43, 2.14) | 53 | 271 | 1.87 (1.38, 2.53) | 1.67 (1.22, 2.29) | 67 | 358 | 1.82 (1.39, 2.37) | 1.77 (1.35, 2.32) |
| 50-59 | Nephrolithiasis |  |  |  |  |  |  |  |  |  |  |  |  |
|  | No | 2468 | 23172 | 1 (Ref) | 1 (Ref) | 1041 | 9871 | 1 (Ref) | 1 (Ref) | 1427 | 13301 | 1 (Ref) | 1 (Ref) |
|  | Yes | 130 | 746 | 1.65 (1.36, 2.00) | 1.48 (1.22, 1.81) | 57 | 321 | 1.71 (1.27, 2.29) | 1.48 (1.09, 2.01) | 73 | 425 | 1.61 (1.25, 2.08) | 1.52 (1.18, 1.97) |
| 60-69 | Nephrolithiasis |  |  |  |  |  |  |  |  |  |  |  |  |
|  | No | 1723 | 15020 | 1 (Ref) | 1 (Ref) | 720 | 6286 | 1 (Ref) | 1 (Ref) | 1003 | 8734 | 1 (Ref) | 1 (Ref) |
|  | Yes | 102 | 547 | 1.61 (1.30, 2.01) | 1.50 (1.20, 1.87) | 45 | 231 | 1.71 (1.23, 2.39) | 1.63 (1.16, 2.31) | 57 | 316 | 1.54 (1.15, 2.07) | 1.46 (1.09, 1.96) |
| 70-85 | Nephrolithiasis |  |  |  |  |  |  |  |  |  |  |  |  |
|  | No | 1108 | 8340 | 1 (Ref) | 1 (Ref) | 493 | 3755 | 1 (Ref) | 1 (Ref) | 615 | 4585 | 1 (Ref) | 1 (Ref) |
|  | Yes | 76 | 285 | 1.96 (1.51, 2.56) | 1.73 (1.31, 2.28) | 31 | 114 | 2.01 (1.32, 3.05) | 1.61 (1.01, 2.57) | 45 | 171 | 1.94 (1.37, 2.73) | 1.80 (1.27, 2.55) |
| **Sex** |  |  |  |  |  |  |  |  |  |  |  |  |  |
| Male | Nephrolithiasis |  |  |  |  |  |  |  |  |  |  |  |  |
|  | No | 6189 | 59180 | 1 (Ref) | 1 (Ref) | 2711 | 26006 | 1 (Ref) | 1 (Ref) | 3478 | 33174 | 1 (Ref) | 1 (Ref) |
|  | Yes | 356 | 1902 | 1.74 (1.55, 1.96) | 1.62 (1.44, 1.83) | 159 | 814 | 1.85 (1.55, 2.21) | 1.72 (1.43, 2.07) | 197 | 1,088 | 1.66 (1.42, 1.95) | 1.58 (1.35, 1.85) |
| Female | Nephrolithiasis |  |  |  |  |  |  |  |  |  |  |  |  |
|  | No | 4219 | 37440 | 1 (Ref) | 1 (Ref) | 1771 | 15794 | 1 (Ref) | 1 (Ref) | 2448 | 21646 | 1 (Ref) | 1 (Ref) |
|  | Yes | 140 | 689 | 1.79 (1.48, 2.15) | 1.59 (1.32, 1.92) | 56 | 282 | 1.73 (1.30, 2.32) | 1.38 (1.01, 1.88) | 84 | 407 | 1.82 (1.43, 2.32) | 1.72 (1.35, 2.19) |
| **Residential location** |  |  |  |  |  |  |  |  |  |  |  |  |  |
| 1. Stockholm | Nephrolithiasis |  |  |  |  |  |  |  |  |  |  |  |  |
|  | No | 2205 | 20447 | 1 (Ref) | 1 (Ref) | 709 | 6639 | 1 (Ref) | 1 (Ref) | 1496 | 13808 | 1 (Ref) | 1 (Ref) |
|  | Yes | 87 | 524 | 1.54 (1.22, 1.94) | 1.44 (1.14, 1.83) | 33 | 180 | 1.75 (1.19, 2.58) | 1.67 (1.12, 2.51) | 54 | 344 | 1.43 (1.07, 1.92) | 1.36 (1.01, 1.82) |
| 2. Uppsala-Orebro | Nephrolithiasis |  |  |  |  |  |  |  |  |  |  |  |  |
|  | No | 2215 | 20851 | 1 (Ref) | 1 (Ref) | 1049 | 9855 | 1 (Ref) | 1 (Ref) | 1166 | 10996 | 1 (Ref) | 1 (Ref) |
|  | Yes | 110 | 529 | 1.94 (1.57, 2.40) | 1.81 (1.46, 2.25) | 47 | 231 | 1.90 (1.38, 2.63) | 1.73 (1.23, 2.43) | 63 | 298 | 1.97 (1.49, 2.62) | 1.90 (1.43, 2.52) |
| 3. West | Nephrolithiasis |  |  |  |  |  |  |  |  |  |  |  |  |
|  | No | 1811 | 16778 | 1 (Ref) | 1 (Ref) | 810 | 7536 | 1 (Ref) | 1 (Ref) | 1001 | 9242 | 1 (Ref) | 1 (Ref) |
|  | Yes | 84 | 388 | 2.01 (1.58, 2.56) | 1.79 (1.40, 2.30) | 39 | 183 | 2.01 (1.41, 2.87) | 1.72 (1.18, 2.51) | 45 | 205 | 2.01 (1.44, 2.79) | 1.86 (1.33, 2.60) |
| 4. South | Nephrolithiasis |  |  |  |  |  |  |  |  |  |  |  |  |
|  | No | 1852 | 17001 | 1 (Ref) | 1 (Ref) | 868 | 8066 | 1 (Ref) | 1 (Ref) | 984 | 8935 | 1 (Ref) | 1 (Ref) |
|  | Yes | 91 | 513 | 1.60 (1.27, 2.01) | 1.39 (1.09, 1.76) | 46 | 232 | 1.83 (1.32, 2.55) | 1.50 (1.06, 2.13) | 45 | 281 | 1.41 (1.02, 1.95) | 1.28 (0.92, 1.79) |
| 5. Southeast | Nephrolithiasis |  |  |  |  |  |  |  |  |  |  |  |  |
|  | No | 1202 | 11241 | 1 (Ref) | 1 (Ref) | 588 | 5486 | 1 (Ref) | 1 (Ref) | 614 | 5755 | 1 (Ref) | 1 (Ref) |
|  | Yes | 77 | 339 | 2.12 (1.64, 2.74) | 2.00 (1.53, 2.60) | 33 | 150 | 2.07 (1.40, 3.07) | 1.97 (1.31, 2.97) | 44 | 189 | 2.16 (1.54, 3.04) | 2.07 (1.47, 2.92) |
| 6. North | Nephrolithiasis |  |  |  |  |  |  |  |  |  |  |  |  |
|  | No | 1123 | 10302 | 1 (Ref) | 1 (Ref) | 458 | 4218 | 1 (Ref) | 1 (Ref) | 665 | 6084 | 1 (Ref) | 1 (Ref) |
|  | Yes | 47 | 298 | 1.42 (1.04, 1.95) | 1.37 (0.99, 1.88) | 17 | 120 | 1.26 (0.75, 2.13) | 1.18 (0.69, 2.03) | 30 | 178 | 1.53 (1.03, 2.27) | 1.51 (1.02, 2.25) |

*Odds ratios adjusted for history of autoimmune diseases, renal insufficiency or failure, metabolic diseases, and osteoporosis.

^†^Sarcoidosis was defined as severe if the patient received immunosuppressive treatment within ± 3 months of diagnosis.

**Table S4:** Odds ratios and 95% confidence intervals (OR 95% CI) for the association between history of nephrolithiasis and risk of sarcoidosis when conducting sensitivity analyses, which adjusted for renal failure/insufficiency, metabolic syndrome related diseases and osteoporosis only if they occurred at least 5 years before the index date, and before nephrolithiasis diagnosis. General population controls were matched to sarcoidosis patients on age, sex, calendar year and county of residence.

|  | **Sarcoidosis cases vs Controls** | | | | **Severe sarcoidosis**^†^ **vs Controls** | | | | **Non-severe sarcoidosis vs Controls** | | | |
| --- | --- | --- | --- | --- | --- | --- | --- | --- | --- | --- | --- | --- |
|  | **Cases N** | **Controls N** | **OR (95% CI)** | **Adjusted OR* (95% CI)** | **Cases N** | **Controls N** | **OR (95% CI)** | **Adjusted OR* (95% CI)** | **Cases N** | **Controls N** | **OR (95% CI)** | **Adjusted OR* (95% CI)** |
| Nephrolithiasis |  |  |  |  |  |  |  |  |  |  |  |  |
| No | 10408 | 96620 | 1 (Ref) | 1 (Ref) | 4482 | 41800 | 1 (Ref) | 1 (Ref) | 5926 | 54820 | 1 (Ref) | 1 (Ref) |
| Yes | 496 | 2591 | 1.75 (1.59, 1.94) | 1.74 (1.57, 1.92) | 215 | 1096 | 1.82 (1.56, 2.11) | 1.81 (1.55, 2.10) | 281 | 1495 | 1.71 (1.50, 1.95) | 1.69 (1.49, 1.93) |
| Severe Nephrolithiasis** |  |  |  |  |  |  |  |  |  |  |  |  |
| No | 419 | 2188 | 1 (Ref) | insufficient | 180 | 936 | 1 (Ref) | insufficient | 239 | 1252 | 1 (Ref) | insufficient |
| Yes | 77 | 403 | 1.13 (0.61, 2.11) | counts | 35 | 160 | 1.35 (0.58, 3.16) | counts | 42 | 243 | 0.93 (0.38, 2.31) | counts |

*Odds ratios adjusted for history of autoimmune disease (any time before index date), and for history of renal insufficiency or failure, metabolic diseases, and osteoporosis (if they occurred more than 5 years before the index date). Diagnoses recorded more than 5 years before index date, but after nephrolithiasis, were not considered as confounders in these models.

^†^Sarcoidosis was defined as severe if the patient received immunosuppressive treatment within ± 3 months of diagnosis.

**Severe nephrolithiasis was defined as meeting at least one of the following criteria: (1) intervention with percutaneous nephrolithotomy (PNCL); (2) multiple stones, defined as >1 stone during a 5-year period, with every stone being >1 year apart; (3) stone formation before age 25; (4) prescription of potassium citrate.

**Table S5:** Odds ratios and 95% confidence intervals (OR 95% CI), stratified by age at the index date, sex and residential location, for the association between history of nephrolithiasis and risk of sarcoidosis when conducting sensitivity analyses, which adjusted for renal failure/insufficiency, metabolic diseases and osteoporosis only if they occurred at least 5 years before the index date, and before nephrolithiasis diagnosis. General population controls were matched to sarcoidosis patients on age, sex, calendar year and county of residence.

|  | | **Sarcoidosis vs Controls** | | | | **Severe sarcoidosis**^†^ **vs Controls** | | | | **Non-severe sarcoidosis cases vs Controls** | | | |
| --- | --- | --- | --- | --- | --- | --- | --- | --- | --- | --- | --- | --- | --- |
|  |  | **Cases N** | **Controls N** | **OR (95% CI)** | **Adjusted OR* (95% CI)** | **Cases N** | **Controls N** | **OR (95% CI)** | **Adjusted OR* (95% CI)** | **Cases N** | **Controls N** | **OR (95% CI)** | **Adjusted OR* (95% CI)** |
| **Age at the index date** |  |  |  |  |  |  |  |  |  |  |  |  |  |
| 18-39 | Nephrolithiasis |  |  |  |  |  |  |  |  |  |  |  |  |
|  | No | 2646 | 26020 | 1 (Ref) | 1 (Ref) | 1203 | 11889 | 1 (Ref) | 1 (Ref) | 1443 | 14131 | 1 (Ref) | 1 (Ref) |
|  | Yes | 68 | 384 | 1.73 (1.33, 2.25) | 1.72 (1.32, 2.24) | 29 | 159 | 1.79 (1.20, 2.69) | 1.76 (1.18, 2.65) | 39 | 225 | 1.69 (1.19, 2.39) | 1.69 (1.19, 2.39) |
| 40-49 | Nephrolithiasis |  |  |  |  |  |  |  |  |  |  |  |  |
|  | No | 2463 | 24068 | 1 (Ref) | 1 (Ref) | 1025 | 9999 | 1 (Ref) | 1 (Ref) | 1438 | 14069 | 1 (Ref) | 1 (Ref) |
|  | Yes | 120 | 629 | 1.84 (1.50, 2.25) | 1.82 (1.49, 2.23) | 53 | 271 | 1.87 (1.38, 2.53) | 1.86 (1.37, 2.52) | 67 | 358 | 1.82 (1.39, 2.37) | 1.80 (1.37, 2.35) |
| 50-59 | Nephrolithiasis |  |  |  |  |  |  |  |  |  |  |  |  |
|  | No | 2468 | 23172 | 1 (Ref) | 1 (Ref) | 1041 | 9871 | 1 (Ref) | 1 (Ref) | 1427 | 13301 | 1 (Ref) | 1 (Ref) |
|  | Yes | 130 | 746 | 1.65 (1.36, 2.00) | 1.63 (1.34, 1.97) | 57 | 321 | 1.71 (1.27, 2.29) | 1.69 (1.26, 2.27) | 73 | 425 | 1.61 (1.25, 2.08) | 1.60 (1.24, 2.06) |
| 60-69 | Nephrolithiasis |  |  |  |  |  |  |  |  |  |  |  |  |
|  | No | 1723 | 15020 | 1 (Ref) | 1 (Ref) | 720 | 6286 | 1 (Ref) | 1 (Ref) | 1003 | 8734 | 1 (Ref) | 1 (Ref) |
|  | Yes | 102 | 547 | 1.61 (1.30, 2.01) | 1.61 (1.29, 2.00) | 45 | 231 | 1.71 (1.23, 2.39) | 1.72 (1.23, 2.40) | 57 | 316 | 1.54 (1.15, 2.07) | 1.54 (1.15, 2.06) |
| 70-85 | Nephrolithiasis |  |  |  |  |  |  |  |  |  |  |  |  |
|  | No | 1108 | 8340 | 1 (Ref) | 1 (Ref) | 493 | 3755 | 1 (Ref) | 1 (Ref) | 615 | 4585 | 1 (Ref) | 1 (Ref) |
|  | Yes | 76 | 285 | 1.96 (1.51, 2.56) | 1.97 (1.51, 2.58) | 31 | 114 | 2.01 (1.32, 3.05) | 2.04 (1.34, 3.12) | 45 | 171 | 1.94 (1.37, 2.73) | 1.93 (1.37, 2.74) |
| **Sex** |  |  |  |  |  |  |  |  |  |  |  |  |  |
| Male | Nephrolithiasis |  |  |  |  |  |  |  |  |  |  |  |  |
|  | No | 6189 | 59180 | 1 (Ref) | 1 (Ref) | 2711 | 26006 | 1 (Ref) | 1 (Ref) | 3478 | 33174 | 1 (Ref) | 1 (Ref) |
|  | Yes | 356 | 1902 | 1.74 (1.55, 1.96) | 1.74 (1.54, 1.95) | 159 | 814 | 1.85 (1.55, 2.21) | 1.86 (1.56, 2.22) | 197 | 1088 | 1.66 (1.42, 1.95) | 1.65 (1.41, 1.93) |
| Female | Nephrolithiasis |  |  |  |  |  |  |  |  |  |  |  |  |
|  | No | 4219 | 37440 | 1 (Ref) | 1 (Ref) | 1771 | 15794 | 1 (Ref) | 1 (Ref) | 2448 | 21646 | 1 (Ref) | 1 (Ref) |
|  | Yes | 140 | 689 | 1.79 (1.48, 2.15) | 1.74 (1.45, 2.10) | 56 | 282 | 1.73 (1.30, 2.32) | 1.68 (1.25, 2.25) | 84 | 407 | 1.82 (1.43, 2.32) | 1.79 (1.41, 2.28) |
| **Residential location** |  |  |  |  |  |  |  |  |  |  |  |  |  |
| 1. Stockholm | Nephrolithiasis |  |  |  |  |  |  |  |  |  |  |  |  |
|  | No | 2205 | 20447 | 1 (Ref) | 1 (Ref) | 709 | 6639 | 1 (Ref) | 1 (Ref) | 1496 | 13808 | 1 (Ref) | 1 (Ref) |
|  | Yes | 87 | 524 | 1.54 (1.22, 1.94) | 1.53 (1.21, 1.94) | 33 | 180 | 1.75 (1.19, 2.58) | 1.80 (1.22, 2.65) | 54 | 344 | 1.43 (1.07, 1.92) | 1.42 (1.06, 1.90) |
| 2. Uppsala-Orebro | Nephrolithiasis |  |  |  |  |  |  |  |  |  |  |  |  |
|  | No | 2215 | 20851 | 1 (Ref) | 1 (Ref) | 1049 | 9855 | 1 (Ref) | 1 (Ref) | 1166 | 10996 | 1 (Ref) | 1 (Ref) |
|  | Yes | 110 | 529 | 1.94 (1.57, 2.40) | 1.94 (1.57, 2.40) | 47 | 231 | 1.90 (1.38, 2.63) | 1.92 (1.39, 2.65) | 63 | 298 | 1.97 (1.49, 2.62) | 1.96 (1.48, 2.60) |
| 3. West | Nephrolithiasis |  |  |  |  |  |  |  |  |  |  |  |  |
|  | No | 1811 | 16778 | 1 (Ref) | 1 (Ref) | 810 | 7536 | 1 (Ref) | 1 (Ref) | 1001 | 9242 | 1 (Ref) | 1 (Ref) |
|  | Yes | 84 | 388 | 2.01 (1.58, 2.56) | 1.98 (1.55, 2.53) | 39 | 183 | 2.01 (1.41, 2.87) | 1.99 (1.39, 2.85) | 45 | 205 | 2.01 (1.44, 2.79) | 1.99 (1.43, 2.77) |
| 4. South | Nephrolithiasis |  |  |  |  |  |  |  |  |  |  |  |  |
|  | No | 1852 | 17001 | 1 (Ref) | 1 (Ref) | 868 | 8066 | 1 (Ref) | 1 (Ref) | 984 | 8935 | 1 (Ref) | 1 (Ref) |
|  | Yes | 91 | 513 | 1.60 (1.27, 2.01) | 1.56 (1.23, 1.96) | 46 | 232 | 1.83 (1.32, 2.55) | 1.78 (1.28, 2.48) | 45 | 281 | 1.41 (1.02, 1.95) | 1.38 (0.99, 1.91) |
| 5. Southeast | Nephrolithiasis |  |  |  |  |  |  |  |  |  |  |  |  |
|  | No | 1202 | 11241 | 1 (Ref) | 1 (Ref) | 588 | 5486 | 1 (Ref) | 1 (Ref) | 614 | 5755 | 1 (Ref) | 1 (Ref) |
|  | Yes | 77 | 339 | 2.12 (1.64, 2.74) | 2.13 (1.64, 2.75) | 33 | 150 | 2.07 (1.40, 3.07) | 2.10 (1.42, 3.12) | 44 | 189 | 2.16 (1.54, 3.04) | 2.17 (1.54, 3.05) |
| 6. North | Nephrolithiasis |  |  |  |  |  |  |  |  |  |  |  |  |
|  | No | 1123 | 10302 | 1 (Ref) | 1 (Ref) | 458 | 4218 | 1 (Ref) | 1 (Ref) | 665 | 6084 | 1 (Ref) | 1 (Ref) |
|  | Yes | 47 | 298 | 1.42 (1.04, 1.95) | 1.40 (1.02, 1.92) | 17 | 120 | 1.26 (0.75, 2.13) | 1.23 (0.73, 2.08) | 30 | 178 | 1.53 (1.03, 2.27) | 1.52 (1.02, 2.26) |

*Odds ratios adjusted for history of autoimmune disease (any time before index date), and for history of renal insufficiency or failure, metabolic diseases, and osteoporosis (if they occurred more than 5 years before the index date). Diagnoses recorded more than 5 years before index date, but after nephrolithiasis, were not considered as confounders in these models.

^†^Sarcoidosis was defined as severe if the patient received immunosuppressive treatment within ± 3 months of diagnosis.
